# Lysosomal Channels as New Molecular Targets in the Pharmacological Therapy of Neurodegenerative Diseases *via* Autophagy Regulation

**DOI:** 10.2174/1570159X22666240517101846

**Published:** 2024-05-17

**Authors:** Valentina Tedeschi, Silvia Sapienza, Raffaella Ciancio, Lorella Maria Teresa Canzoniero, Anna Pannaccione, Agnese Secondo

**Affiliations:** 1 Department of Neuroscience, Reproductive and Odontostomatological Sciences, Federico II University of Naples, *Via* S. Pansini 5, 80131 Naples, Italy;; 2 Department of Science and Technology-DST, University of Sannio, Via Port'Arsa 11, 82100, Benevento, Italy

**Keywords:** Lysosomal channels, TRPML1, TPC2, autophagy, neurodegenerative diseases, AD, PD, HD, ALS, LSD, TFEB

## Abstract

Besides controlling several organellar functions, lysosomal channels also guide the catabolic “self-eating” process named autophagy, which is mainly involved in protein and organelle quality control. Neuronal cells are particularly sensitive to the rate of autophagic flux either under physiological conditions or during the degenerative process. Accordingly, neurodegeneration occurring in Parkinson’s (PD), Alzheimer’s (AD), and Huntington's Diseases (HD), and Amyotrophic Lateral Sclerosis (ALS) as well as Lysosomal Storage Diseases (LSD) is partially due to defective autophagy and accumulation of toxic aggregates. In this regard, dysfunction of lysosomal ionic homeostasis has been identified as a putative cause of aberrant autophagy. From a therapeutic perspective, Transient Receptor Potential Channel Mucolipin 1 (TRPML1) and Two-Pore Channel isoform 2 (TPC2), regulating lysosomal homeostasis, are now considered promising druggable targets in neurodegenerative diseases. Compelling evidence suggests that pharmacological modulation of TRPML1 and TPC2 may rescue the pathological phenotype associated with autophagy dysfunction in AD, PD, HD, ALS, and LSD. Although pharmacological repurposing has identified several already used drugs with the ability to modulate TPC2, and several tools are already available for the modulation of TRPML1, many efforts are necessary to design and test new entities with much higher specificity in order to reduce dysfunctional autophagy during neurodegeneration.

## 1 INTRODUCTION

Lysosomal dysfunction has been associated with different neurodegenerative disorders, thereby causing an impairment of the catabolic “self-eating” process named autophagy [1, 2]. This is possibly due to the strong dependency of neurons on this cleansing mechanism mainly involved in protein and organelle quality control. In this respect, defective autophagy determines the accumulation of autophagic substrates, leading to oxidative stress and the accumulation of toxic aggregates. Therefore, lysosomal dysfunction and abnormal autophagic flux are now considered very closely related mechanisms involved in neurodegeneration [3]. This has been exemplified in Parkinson’s (PD), Alzheimer’s (AD), and Huntington's Disease (HD), as well as Amyotrophic Lateral Sclerosis (ALS). Accordingly, besides their clinicopathologically distinct characteristics, they share, as a common pathomechanism, iterative protein aggregation in those brain regions undergoing neurodegeneration. For instance, the accumulation of aggregates of amyloid-beta and tau in AD, alpha-synuclein in PD and other synucleinopathies, and TAR DNA-binding Protein 43 (TDP-43) in ALS has been largely associated with the progressive neurodegeneration and clinical symptoms [4, 5]. At the genetic level, genome-wide association studies show at least some degree of genetic correlation among AD, PD, Frontotemporal Dementia (FTD), and ALS [6-8]. Therefore, lysosomal/autophagic dysfunction represents one of the main transdiagnostic processes underlying multiple neurodegenerative disorders [9, 10]. Of interest, new evidence indicates a strict association among air pollution nanoparticles, lysosomal dysfunction, and neurodegeneration [11-13].

By controlling organelle viability and several other cell functions, lysosome is now considered an important Ca^2+^ store with its ionic machinery. For instance, Vacuolar-type H^+^-ATPases (V-ATPases) maintain the acidic pH by pumping protons into the lysosomal lumen [14], playing an important role in osteoclast bone resorption [15], regulation of systemic pH in the kidney [16], and cancer cells invasion [17].

Moreover, lysosomal ion homeostasis in mammals is regulated by several channel families: Transient Receptor Potential Channel Mucolipins (TRPMLs), composed of the three members TRPML1, TRPML2, and TRPML3; the Two-Pore Channels (TPCs), composed of two members, TPC1 and TPC2; the Ca^2+^–activated Big-conductance K^+^ channels (BK), the Transmembrane Protein 175 (TMEM175) channel, and the CLC family of chloride (Cl^-^) channels and chloride (Cl^-^)/proton (H+) transporters whose best-characterized member is CLC-7 [18]. Of note, most of these ionic proteins cooperate to regulate themselves and lysosomal functions downstream. This is exemplified by BK channels that couple with TRPML1 to regulate lysosomal Ca^2+^ homeostasis through a fine modulation of TRPML1 function [19, 20] and lysosomal membrane trafficking [21, 22]. Furthermore, in addition to lysosomal pH, the Cl− channel CLN7 also regulates lysosomal Ca^2+^ content through the control of TRPML1 [23]. Functionally, TRPML and TPC channel families are non-redundant and they are involved in the modulation of mutually exclusive processes, including activation of the Transcription Factor EB (TFEB) [24], lysosomal volume [25], choroidal angiogenesis [26], and oxytocin neurosecretion [27].

## 2 TFEB-INDUCED LYSOSOMAL BIOGENESIS AS MOLECULAR DETERMINANT OF AUTOPHAGY

The Transcription Factor EB (TFEB) is now considered the master regulator of autophagy through the expression of CLEAR motif-containing target genes mainly involved in lysosomal biogenesis [28-30].

Therefore, TFEB is essential for the formation of autophagosomes and fusion with lysosomes, thereby facilitating substrate clearance and lysosomal exocytosis [31], a neuroprotective process by which these tiny organelles can secrete their content out by fusing themselves to the cell membrane [32, 33].

Interestingly, enhancing lysosomal biogenesis, TFEB overexpression allows the degradation of damaged organelles, lipid droplets, and protein aggregates, thus alleviating specific neurodegenerative phenotypes in preclinical models of Parkinson’s and Alzheimer’s diseases [34].

**Fig. (1):**
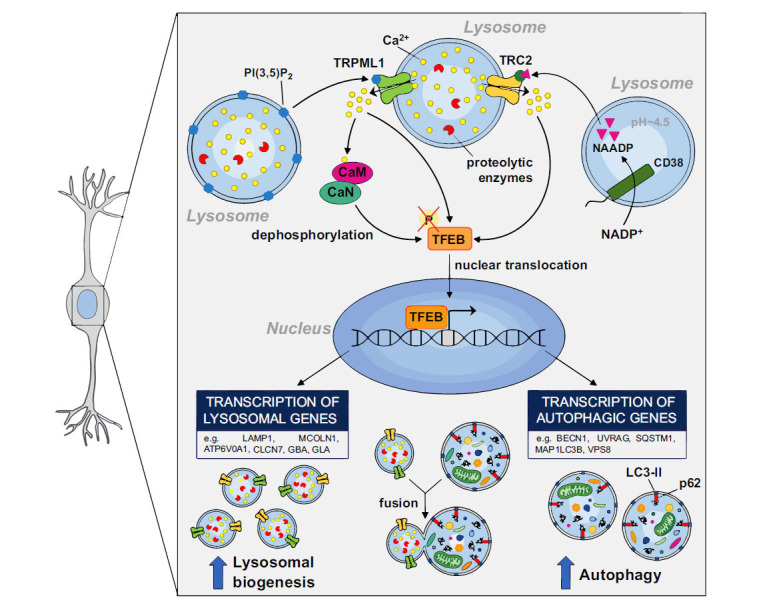
Mechanism of the transcription factor EB (TFEB) activation and translocation by TRPML1 and TPC2 Ca^2+^ release. Schematic representation of TFEB activation by calcium (Ca^2+^) release from lysosomal channels TRPML1 and TPC2. Upon TRPML1 activation, Ca^2+^ induces the activation of Calcineurin (CaN), a unique Ca^2+^-and calmodulin (CaM)-dependent serine/threonine phosphatase that promotes stimulation of TFEB by dephosphorylation. Then, nuclear TFEB translocation promotes the transcription of lysosomal and autophagy genes.

Mechanistically, TFEB activation, with the consequent transcriptional stimulation of lysosomal and autophagic genes, is due to its dephosphorylation by the phosphatase calcineurin mainly triggered by Ca^2+^ released through TRPML1 [24, 35] (Fig. **1**). Consistently, silencing of TFEB prevents TRPML1-dependent modulation of autophagy, causing Ca^2+^ accumulation in the lumen of late endosomes [36, 37]. Interestingly, TRPML1 can modulate autophagy in a rapid way, by promoting autophagic vesicle and lysosome fusion events through the activation of the complex calcium/ calmodulin-dependent protein kinase kinase (CaMKKβ)/ ULK1/hVPS34, and in a sustained way, by inducing TFEB nuclear translocation [38]. In addition to the prominent role of TRPML1 in autophagy regulation, this channel is involved in maintaining the acidic milieu of the lysosome, thereby ensuring the proper functioning of degradative enzymes [39]. Of interest, post-mortem brain tissue from ALS patients expressed a reduced nuclear localization of TFEB, suggesting that the lysosome-autophagy pathway is impaired during neurodegenerative diseases [40].

## 3 LYSOSOMAL CHANNEL TRPML1 AS MOLECULAR REGULATOR OF AUTOPHAGY *VIA* Ca^2+^ RELEASE DURING NEURODEGENERATION

**Fig. (2):**
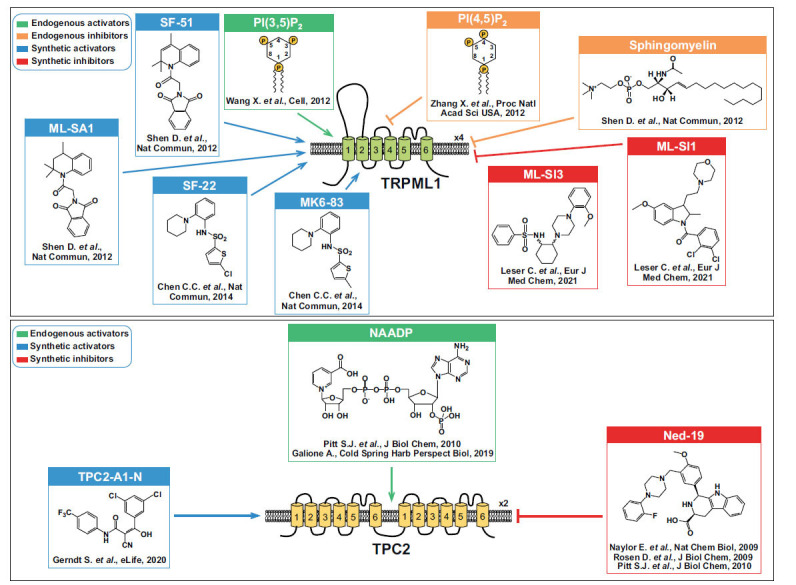
Pharmacological modulation of TRPML1 and TPC2. TRPML1 modulators (either stimulators or inhibitors) have been reported in the upper part of the scheme. The structure of the TRPML1 endogenous agonist, PI(3,5)P2, has been depicted in green. TPC2 modulators inducing Ca^2+^-release have been reported at the bottom. The structure of the TPC2 endogenous agonist, NAADP, has been depicted in green.

MCOLN1/TRPML1 is mutated in Mucolipidosis type IV (MLIV: OMIM 252650) [41], an autosomal recessive LSD characterized by delayed psychomotor development, corneal opacities and neurodegeneration. This neurological symptomatology is due to defective lysosome-associated pathways, including autophagy [42-45]. Pharmacological stimulation of TRPML1 by synthetic agonists determines the activation of autophagy through the rapid induction of LC3 puncta formation and autophagic vesicle-lysosome fusion events (Fig. **2**).

From a therapeutic perspective, TRPML1 is now considered an interesting pharmacological target against neurodegeneration. Of note, TRPML1 activation rescues dysfunctional phenotype in lysosomal storage diseases, including Neuronal Ceroid Lipofuscinoses (NCL, Batten disease), mucopolysaccharidoses, such as Hurler or Hunter syndrome, sphingolipidoses, such as Fabry, Gaucher or Niemann–Pick-type C1 (NPC1), and mucolipidoses, such as Mucolipidosis type IV (MLIV), characterized by a greater neurodegenerative component [46-51].

Although, only recently, TRPML1 has been assumed to play an important role as a pharmacological target able to modulate autophagy in other neurodegenerative diseases, including AD, PD, and ALS.

### 3.1 TRPML1 In ALS

Lysosomal ionic dyshomeostasis, due to the reduced function of lysosomal channels, has been shown to be involved in both familial and sporadic forms of ALS. Accordingly, mutations of FIG4 impairing biosynthesis of PI(3,5)P_2_, the endogenous ligand of endolysosomal TRPML channels, can cause the rare autosomal recessive Charcot-Marie-Tooth peripheral neuropathy type 4 J (CMT4J) and have been implicated also in familial amyotrophic lateral sclerosis (ALS) type 11 [52]. In fibroblasts from FIG4 deficient patients, the abnormal level of intralysosomal Ca^2+^ and lysosomal storage function has been rescued by the pharmacological activation of TRPML1 [53].

Furthermore, ML-SA1-induced lysosomal Ca^2+^ release by TRPML1 is able to promote a sort of autophagy reprogramming, leading to a long-lasting effect on motor neurons exposed to β-methylamino-L-alanine (L-BMAA), a neurotoxin reproducing Amyotrophic Lateral Sclerosis and Parkinsonism-Dementia Complex (ALS-PDC), a disease’s type of apparent environmental origin [54].

### 3.2 TRPML1 In PD

The Transcription Factor EB (TFEB) overexpression stimulates autophagy in midbrain dopaminergic neurons, thus preventing neurotoxicity induced by α-synuclein (α-syn) accumulation [55]. In accordance with these results, Tsunemi *et al.* showed that synuclein accumulation could be cleared *via* lysosomal exocytosis induced by TRPML1 stimulation in PD dopaminergic neurons [56]. A recent study has confirmed the neuroprotective role of TRPML1 in PD *via* autophagy induction [57]. Accordingly, Artemisia leaf extract could exert neuroprotection against MPP^+^-mediated PD in SH-SY5Y cells and a PD mouse model by inducing TRPML1 upregulation and boosting autophagy/mitophagy. This triggers a protective cascade counteracting mitochondrial damage, ROS generation, and α-synuclein accumulation. However, not only neurons, but also glial cells, are involved in the protective effect of restored autophagy in PD. In fact, when autophagic function has been enhanced in microglial cells, α-syn is degraded, thus boosting therapeutic efficacy in PD [58].

### 3.3 TRPML1 In HD

The basis for autophagic impairment in HD involves the complex relationship between huntingtin and the autophagic receptors highly expressed in huntingtin aggregates [59]. This suggests the occurrence of autophagy deficiency in the pathology and a direct toxic effect of mutated huntingtin itself [60]. Accordingly, the polyglutamine tract of mutant huntingtin competes with beclin-1, preventing the normal rate of autophagy [61]. Moreover, no evidence of the direct involvement of TRPML1 has been reported yet.

### 3.4 TRPML1 In AD

In a preclinical model of AD, such as APP/PS1 transgenic mice, TRPML1 is downregulated at the neuronal level, determining beclin-1 and LC3 protein upregulation as a sign of autophagy dysfunction and apoptosis [62]. This testifies the impairment of lysosomal-dependent protein quality control during the neurodegenerative process.

Moreover, in a triple transgenic gp120/APP/PS1 mouse, β-amyloid peptides were reduced by pharmacological activation of TRPML1, promoting deposit clearance through lysosomal Ca^2+^ efflux [63].

Furthermore, TRPML1 agonist ML-SA1 reduced LDL-induced amyloidogenesis in the AD brain, while TRPML1 silencing potentiated the detrimental process induced by LDL derived from the peripheral circulation [64].

In accordance with prevailing evidence, iPSC-derived human cortical neurons expressing APOE ε4, a genetic risk factor for late-onset AD, showed a diminished ability of TRPML1 to release Ca^2+^ and a greater accumulation of autophagic vesicles. In this setting, neurons were rescued by TRPML1 stimulation *via* its synthetic agonist ML-SA1 [65].

## 4 LYSOSOMAL CHANNEL TPC2 AS MOLECULAR REGULATOR OF AUTOPHAGY *VIA* Ca^2+^ RELEASE DURING NEURODEGENERATION

**Fig. (3):**
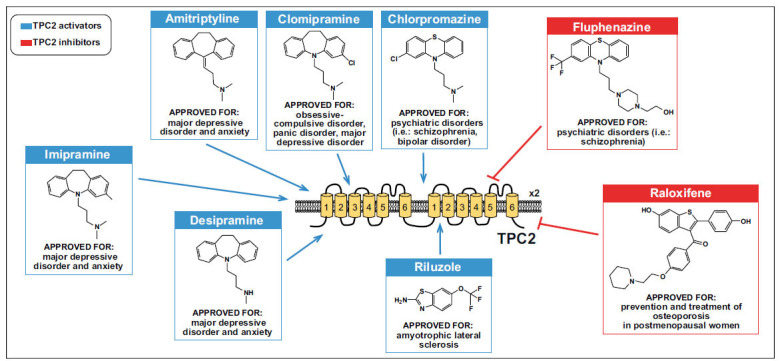
Schematic representation of drug repurposing for TPC2 modulation. Schematic representation of the already used drugs able to modulate the TPC2 channel. Among them, some Tricyclic Anti-depressants (TCAs), including chlorpromazine, clomipramine, desipramine, and amitriptyline, have been reported to be able to stimulate TPC2 as agonists, whereas the typical antipsychotic fluphenazine and the selective estrogen receptor modulator raloxifene have been reported to act as TPC2 antagonists.

Under physiological conditions, TPCs regulate lysosomal functions, including membrane voltage and luminal pH maintenance [66]. Furthermore, Ca^2+^ release from TPC2, due to the stimulation of its endogenous ligand NAADP, is associated with TFEB translocation and autophagy induction [67]. Therefore, in consideration of the occurrence of dysfunctional autophagy in neurodegeneration, targeting the lysosomal TPC2 channel has been suggested as a therapeutic strategy for new drugs’ development in the treatment of neurodegenerative diseases (Fig. **2**). Of note, several pre-approved drugs have been identified for their ability to modulate TPC2 (Fig. **3**). However, a greater effort should be made to identify new entities able to modulate the channel. Moreover, only a few studies are available due to the recent evidence of its involvement.

### 4.1 TPC2 In LSDs

Most of LSDs are characterized by neurodegeneration and its consequences, which include developmental delay, intellectual disability, seizures, motor function, and vision loss. Of note, TPC dysfunction has been associated with LSDs. Furthermore, in NPC1 and other LSDs, TRPML1 activity is also reduced, thereby accumulating sphingomyelin [49]. However, differently from TRPML1, TPC2 activation is pH-independent and not modulated by sphingomyelin, potentially representing a therapeutic advantage over TRPML1 to promote autophagy and aggregates’ degradation. Accordingly, the activation of the Ca^2+^ -permeable TPC2 by synthetic agonists reduces cholesterol or lipofuscin accumulation, abnormal vacuole formation, and neuronal loss [68, 69]. Mechanistically, TPC2-dependent neuroprotection is linked to lysosomal exocytosis and autophagy activation, as assessed in mucolipidosis type IV (MLIV), Niemann-Pick type C1, Batten disease patient fibroblasts, and in iPSC- derived neurons from MLIV and Batten patients [68]. The same level of neuroprotection has been demonstrated in an MLIV mouse model [68], in which TPC2 stimulation reduces astrogliosis and p62 accumulation in the cerebellum and hippocampus, thus restoring motor performance [69].

### 4.2 TPC2 In AD And PD

Amyloid plaques and intraneuronal hyperphosphorylated tau accumulation in AD are due to the impairment of the autophagy-lysosomal pathway [70]. This seems to be partially dependent on the excessive Ca^2+^ release from lysosome through TPC2 gain-of-function. In fact, presenilin 1-expressing SH-SY5Y cells and familial AD fibroblasts display a lysosomal Ca^2+^ leak associated with TPC2 hyperfunctionality [71]. Therefore, genetic knockdown or pharmacological inhibition of this channel restores lysosomal Ca^2+^ homeostasis and promotes reduction in cortical and hippocampal amyloid plaques in AD mice [71]. The same occurred for the neurofibrillary tangle accumulation caused by hyperphosphorylated tau protein whose clearance was restored in Thy1-hTau.P301S transgenic mice by pharmacological inhibition of TPC2, mitigating lysosome alkalinization [72]. Regarding PD, enlarged lysosomal morphology is normalised in LRRK2-PD fibroblasts transfected with a siRNA against TPC2 or treated with pharmacological inhibitors of the channel, thus suggesting a detrimental interaction between TPC2 and LRRK2 during the pathology [73]. Furthermore, lysosomal Ca^2+^ dysregulation and TPC2 malfunction have also been detected in fibroblasts from patients harbouring mutations in the PD-linked gene *GBA1* [74].

### 4.3 TPC2 In Brain Ischemia

Besides the identification of dysfunctional autophagy as one of the main pathomechanisms in stroke [75], only a few studies have focused on TPC2 involvement. The autophagic flux is increased during ischemia as well as during the early phase of reperfusion both in the brain and the heart. Although modulating autophagy is now considered a viable therapeutic strategy for the protection of the brain during ischemia, this process may be differently regulated in each component of the neurovascular unit, including Endothelial Cells (ECs), neurons, or glial cells [76]. For instance, enhanced autophagy is responsible for excessive Blood-Brain Barrier (BBB) disruption during brain ischemia, while autophagy inhibition improves BBB recovery [77]. Of note, at the neuronal level, the pharmacological inhibition of TPC2 rescued primary cortical neurons from hypoxia-induced cell death and reduced the infarct volume in Transient Middle Cerebral Artery Occlusion (tMCAO) mice by blocking autophagy [78]. This highlights the relevance of autophagy modulation as a new therapeutic strategy against stroke.

### 4.4 Drug Repurposing For TPC2

Drug repurposing is a strategy to identify novel uses for pre-approved drugs, and represents one of the most active areas in pharmacology.

Considering the involvement of TPC2 in modulating several cell functions (*i.e*., proliferation, apoptosis, adhesion, invasion, migration, autophagy, and angiogenesis), the putative modulators of this lysosomal channel may be reconsidered for repurposing use (Fig. **3**). In this respect, some tricyclic anti-depressants (TCAs), including chlorpromazine, clomipramine, desipramine, and amitriptyline, which are believed to act on neurotransmitter transporters or voltage-gated Na^+^ channels, are able to stimulate lysosomal TPC2 channel. Moreover, they display synergistic effects with the endogenous ligand PI(3,5)P_2_, inducing a lysosomal Na^+^ current mediated by TPCs. Considering the presence of PI(3,5)P_2_ in the Central Nervous System (CNS), this suggests that TCAs level might be sufficient to cause robust pharmacological actions in the brain through lysosomal activation [79]. On the other hand, some antagonists have also been identified [80]. On the other hand, lysosomes and TPCs have recently been proposed as important modulators of oxytocin secretion, a neuropeptide regulating social behaviour and social interaction [27]. In particular, TPC channels seem to participate in the glutamate-induced secretion of oxytocin by the hypothalamus, once stimulated by its endogenous ligand NAADP [27]. This is interesting evidence suggesting the putative involvement of TPC2 as a pharmacological target of some TCAs.

Moreover, very recently, a relevant involvement of TPCs in cardiac function has been demonstrated. Accordingly, Tpcn1/2^−/−^ mice displayed alterations in the LV systolic and diastolic function, showing a reduction in the amplitude of caffeine-evoked Ca^2+^ transient in cardiomyocytes. On the other hand, a higher TPC expression determined hypertrophy induction [81]. Therefore, it is possible that the use of TPCs agonists in neurodegenerative diseases may influence cardiac function.

## CONCLUSION

Many different neurodegenerative disorders are associated with lysosomal dysfunction and defects in the catabolic “self-eating” process of autophagy. A key role in the induction of the autophagic pathway is played by the lysosomal cation channels TRPML1 and TPC2, mainly involved in the maintenance of lysosomal ionic homeostasis under both physiological and pathological conditions. However, the contribution of these channels in the molecular mechanisms underlying neurodegeneration has only been partially investigated. Thus far, many pharmacological tools have been used to modulate TRPML1 and TPC2 functions in preclinical models of neurodegeneration, in order to restore the impairment in the autophagic flux. However, at present, none of them have been approved for the treatment of neurodegenerative diseases in human patients. A concrete opportunity to accelerate the development of drugs against these disorders could come from drug repurposing, a pharmacological strategy to identify novel uses for pre-approved drugs. Indeed, by using this strategy, some existing drugs, including some tricyclic anti-depressants (TCAs), have been found to modulate the TPC2 channel. Another interesting observation has been the regulation of the lysosomal channels by environmental factors, including air pollution nanoparticles, that have very recently been associated with autophagic dysfunction and neurodegeneration. This issue is largely unexplored. Considering the increasing incidence of neurodegenerative diseases and the high impact of air pollution on human health in many countries, targeting those lysosomal channels responsible for the abnormalities in the autophagic pathway during neurodegeneration could be a valid therapeutic option for the treatment of these disorders. However, the research in the field of pharmacological modulation of lysosomal channels is still in its infancy; therefore, many efforts will be necessary to identify new or pre-approved pharmacological entities able to target these proteins, with the aim to restore the abnormalities in the autophagic pathway observed during neurodegeneration.

## LIST OF ABBREVIATIONS

AD: Alzheimer’s Disease
ALS: Amyotrophic Lateral Sclerosis
BBB: Blood-brain Barrier
CNS: Central Nervous System
ECs: Endothelial Cells
FTD: Frontotemporal Dementia
HD: Huntington's Disease
PD: Parkinson’s Disease
tMCAO: Transient Middle Cerebral Artery Occlusion
